# Supplementary material for: Chromothripsis during telomere crisis is independent of NHEJ, and consistent with a replicative origin
Source: Genome Res. 2019 May;29(5):737–49. doi: 10.1101/gr.240705.118 (PMC6499312; doi:10.1101/gr.240705.118)
Supplement: Supplemental Material [file supp_gr.240705.118_Supplemental_file_1.zip › contigs/annotated_contigs/DB116/contig.2.DB116_length_580_mean_cov_7.91724137931.docx]

**DB116_length_580_mean_cov_7.91724137931**

```
AATTCTGTATGTGTGTGCGTATATAAGCGTTAACCTTAGAACTCGTCACTATACTATTACTTATTTTTAATTATACTTCTTTGCTCACA
 >chr3:21457078-21457273 + E=1e-102
AATCATTTTAGCCCTAATCTTTAAAAATTGCCTTTAGAGATGACCTGCCAGTCTGAAAATAACATGACCCCATTATCGGCTGTCTTAGT

TAACCAAACTCTAAGAT|GATGACGCATTTAAATTGTTGTTCCCTTCCACACCCCCACTGCCAGCTGTCTATTGCCTTTTTCAGGGTTG
                   >chr3:21458356-21458741 + E=5e-214
GTTTTGTTCAGAATTTATTACATTCTTGACTGGGAAAATAGAAGTAGCATATCTTACATGATGTTGGCATAAGTGCGATCATAATAAGT

GAGTACTAAGTGAATTAGCAACTACTCCAATATTGGTGAAATGTACATTCTGGTTGAGCATATGAATGACCTACAATATGGCTTACACT

CAGGATGGAAAGGAATACAGAATTTTTTTCTGGACATATGCAAGTATGTGAGTAGCTGCATTGGTCACTCGAATAAAGAATTTTTATTC

CTGAGAGGATAAAAGCAATGCCCCTCTAGTGCTGATGTATTAAACCA
```
